# HIV deathrate prediction by Gaidai multivariate risks assessment method

**DOI:** 10.1002/iid3.70040

**Published:** 2024-10-16

**Authors:** Oleg Gaidai

**Affiliations:** ^1^ Department of Mechanics and Mathematics Ivan Franko Lviv State University Lviv Ukraine

**Keywords:** AI, AIDS, edidemic outbreak, HIV, mathematical biology, public‐health

## Abstract

**Objectives:**

HIV is a contagious disease with reportedly high transmissibility, being spread worldwide, with certain mortality, allegedly presenting a burden to public health worldwide. The main objective of this study was to determine excessive HIV death risks at any time within any region or country of interest.

**Study design:**

Current study presents a novel multivariate public health system bio‐risk assessment approach that is particularly applicable to environmental multi‐regional, biological, and public health systems, being observed over a representative period of time, yielding reliable long‐term HIV deathrate assessment. Hence, the development of a new bio‐statistical approach, that is, population‐based, multicenter, and medical survey‐based. The expansion of extreme value statistics from the univariate to the bivariate situation meets with numerous challenges. Firstly, the univariate extreme value types theorem cannot be directly extended to the bivariate (2D) case, ‐ not to mention challenges with system dimensionality higher than 2D.

**Methods:**

Existing bio‐statistical methods that process spatiotemporal clinical observations of multinational bio‐processes often do not have the advantage of efficiently dealing with high regional dimensionalities and complex nonlinear inter‐correlations between different national raw datasets. Hence, this study advocates the direct application of the novel bio‐statistical Gaidai method to a raw unfiltered clinical data set.

**Results:**

This investigation described the successful application of a novel bio‐risk assessment approach, yielding reliable long‐term HIV mortality risk assessments.

**Conclusions:**

The suggested risk assessment methodology may be utilized in various public bio and public health clinical applications based on available raw patient survey datasets.

## Key points

- A novel public health bio‐risk assessment methodology was developed and successfully applied to HIV (i.e., Human Immunodeficiency Virus) recorded clinical death rates.
- Disease outbreak's multidimensional spatiotemporal risks estimated.
- Confidence bands assessed for predicted future HIV deathrates.

## Graphical abstract

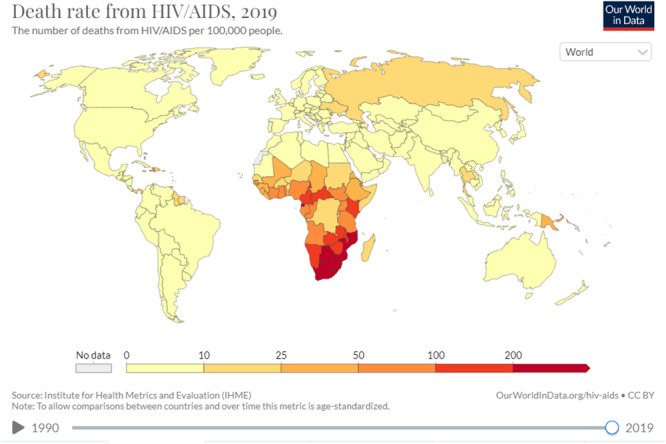

## 1 INTRODUCTION

Biostatistical aspects of HIV or AIDS (Acquired immunodeficiency syndrome), along with other recent similar epidemiological phenomena, have been receiving extensive attention within the modern bio‐research community.^1^, ^2^, ^3^, ^4^, ^5^, ^6^, ^7^, ^8^, ^9^, ^10^, ^11^, ^12^, ^13^, ^14^, ^15^, ^16^, ^17^, ^18^, ^19^ Generally speaking, it is often quite challenging to accurately assess complex biological or public health system risks, along with outbreak risks under realistic epidemic conditions, by using classic theoretical system bio‐risk assessment methods.^20^, ^21^, ^22^, ^23^, ^24^, ^25^ The latter is typically due to the biosystem's high number of degrees of freedom, along with a significant number of stochastic/random variables, governing biological or public health system dynamics, especially when spread over extensive terrains. In general, the bio‐reliability function of complex biological systems may be accurately assessed directly, when sufficient measurements are available, or by extensive MCS (i.e., Monte Carlo Simulations). However, for HIV, the available clinical observation numbers are limited to the year 1990.^26^ Given these arguments, the authors introduced a novel bio‐risk assessment methodology suitable for biological and public health systems for the accurate prediction of epidemic outbreak risks. Hence, this study focused on global HIV epidemics,^20^, ^21^, ^27^ with a specific accent on international cross‐correlations. The global case has been chosen owing to extensive global health observations, along with associated research currently available online.^26^

Bio‐statistical and risk assessment modeling of raw clinical data often relies on EVT (i.e., Extreme Value Theory), which is widely used in medicine and bio‐engineering. Recent studies have argued for and against the life expectancy probability distribution upper bounds.^21^, ^27^ Often studies written within these research fields assume parametric 2D (i.e., bivariate) lifetime distributions, derived from exponential distributions, to match underlying raw clinical datasets.^28^, ^29^, ^30^ In,^31^ the authors proposed a new approach using power variance functional copulas (e.g., Gumbel, inverse Gaussian, Clayton), with conditional samplings being used within survival analysis. In^32^ the authors utilized EVT to predict mutations within evolutionary genetics to determine the effects of mutation fitness. Similarly, in^33^ authors utilized the EVT theory to assess fitness effects, utilizing Beta‐Burr distributions. In^34^ authors discussed the use of a bivariate logistic regression model use to access, for example multiple sclerosis deaths and walking disabilities. Recent studies have used a homogeneous semi‐Markov model to predict HIV epidemic dynamics.^20^ In^28^ authors utilized EVT and GEV (i.e., Generalized Extreme Value) PDF (i.e., Probability Density Function) to estimate future HIV outbreak risks. Using the GEV version of the asymptotic extreme value PDFs to obtain the observed extreme values is a popular method for practical extreme‐value analysis. The extreme values recorded during certain time periods, such as a year, are the usual data employed in contemporary methods such as POT (Peaks‐Over‐the‐Threshold), Generalized Pareto, Gumbel, and other similar methods. Few statistical studies have been conducted to predict HIV or similar contagious disease outbreak risks within a spatiotemporal framework; hence, the proposed novel methodology may provide better insights and indications of possible future spatial spread of various diseases. In the current study, the epidemic outbreak was viewed as an unexpected event or incident that may occur within any region of a given country at any time horizon; hence, spatial spreads have been accounted for. In this study, a special nondimensional factor λ was introduced for use in the prediction of future epidemic risks, at any time horizon, and at any nation/region.

**Figure 1:**
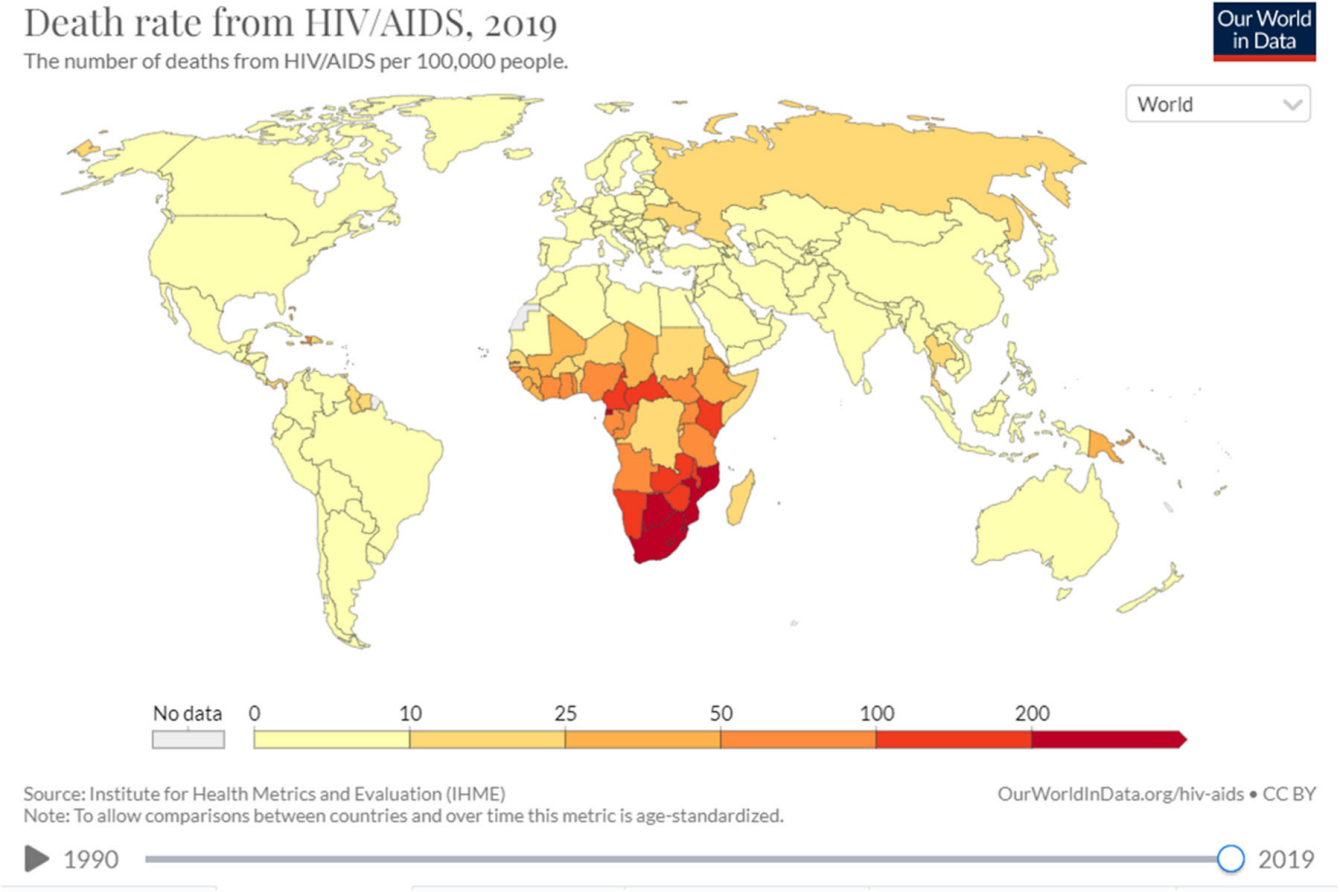
Global map with countries and corresponding HIV‐related death rates. All the world's countries have been studied.^26^

Biological and public health systems are subject to piecewise ergodic and cyclic environmental influences. Another alternative is to model the bio‐process, as it is dependent on a number of specific bio‐environmental parameters whose temporal variation may be modeled by piecewise ergodic processes. The HIV clinical dataset of 195 world countries, during the years 1990–2019 was retrieved from a public website.^26^ This clinical dataset is represented by each world's country, and the bio‐system under consideration may be regarded as a multi‐degree‐of‐freedom MDOF (i.e., Multi‐Degree‐Of‐Freedom) dynamic bio or public health system, with nonlinearly inter‐correlated national/regional dimensions/components. While presented study aimed to reduce the risk of future global epidemic outbreaks by accurately foreseeing them, it has only focused on annually registered patients' death numbers, not on accompanying symptoms. Figure 1 illustrates the global map along with the corresponding HIV‐related death cases.

## 2 GAIDAI SPATIOTEMPORAL RISKS ASSESSMENT METHOD

**Figure 2:**
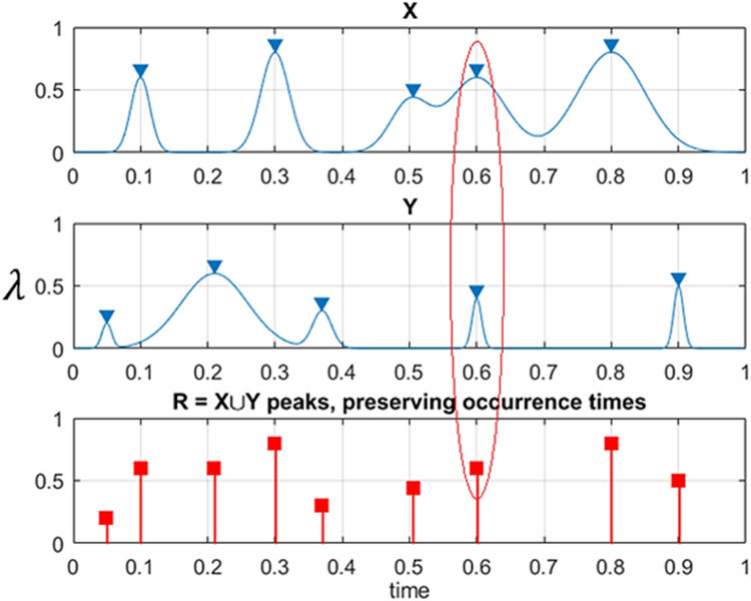
Illustration on how 2 exemplary processes X, Y being combined into 1D synthetic biosystem's vector R. Red ellipse marks case of simultaneous maxima for 2 biosystem's principal components.

The dataset studied herein was collected over three decades between years 1990–2020.^26^ Let us consider the MDOF biosystem being exposed to temporarily stationary random piecewise ergodic biological and/or environmental influences. The biosystem is represented by its critical components, assembled into one biosystem vector(X(t),Y(t),Z(t),…), consisting of the biosystem's principal dimensions/components X(t),Y(t),Z(t),…that have been either simulated/measured/observed over a sufficiently long (i.e., representative) time‐lapse (0,T). The unidimensional (1D) biosystem principal component global maxima to be denoted here as $X_{T}^{\max}=\max_{0\leq t\leq T}X\left(t\right)$, $Y_{T}^{\max}=\max_{0\leq t\leq T}Y\left(t\right)$, 
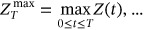
 $Z_{T}^{\max}=\max_{0\leq t\leq T}Z\left(t\right),\ldots \;$. By sufficiently long (i.e., representative) time lapse T authors mean that the value of T is large enough, with respect to the dynamic biosystem's auto‐correlation, as well as relaxation time scales. Let $X_{1},\ldots ,X_{N_{X}}$ be the temporally consequent biosystem critical 1D component bio‐process X=X(t) local maxima, recorded at discrete, temporally increasing time instants $t_{1}^{X}<\cdots <t_{N_{X}}^{X}$ within(0,T). The same definitions follow for other MDOF bio or health system's principal components Y(t),Z(t),… namely $Y_{1},\ldots ,Y_{N_{Y}};$ $Z_{1},\ldots ,Z_{N_{Z}}$ etc. For the sake of simplicity, the local and global maxima of all the biosystem's critical components are assumed to be positive, with

(1) $$P=\overset{\left(\eta_{X},\eta_{Y},\eta_{Z},\;\ldots \right)}{\underset{\left(0,0,0,\;\ldots \right)}{∭}}p_{X_{T}^{\max},Y_{T}^{\max},Z_{T}^{\max},\ldots }\left(x_{T}^{\max},y_{T}^{\max},z_{T}^{\max},\ldots \right)dx_{T}^{\max}dy_{T}^{\max}dz_{T}^{\max}\ldots$$

being target dynamic biosystem's survival chances/probability, given critical/risk/hazard/damage values of biosystem's critical components, denoted here as$\;\eta_{X}$, $\eta_{Y}$, $\eta_{Z}$,…; ∪ being the logical unity operator «or»; $p_{X_{T}^{\max},{\;Y}_{T}^{\max},{\;Z}_{T}^{\max}\;,\ldots }$ being target joint PDF of the individual key component's maxima. When the biosystem's NDOF (i.e., Number of Degrees Of Freedom) becomes large, it may not be feasible to directly assess the target joint PDF $p_{X_{T}^{\max},{\;Y}_{T}^{\max},{\;Z}_{T}^{\max}\;,\ldots }$ and hence the biosystem's target survival probability P. The latter target chance/probability P has to be accurately assessed, as it defines the expected biosystem's lifetime. The biosystem's critical 1D components X,Y,Z,…being re‐scaled, then non‐dimensionalized

(2) $$X\to \frac{X}{\eta_{X}},\;Y\to \frac{Y}{\eta_{Y}},\;Z\to \frac{X}{\eta_{X}},\;\ldots$$

making all biosystems' principal components nondimensional and having the same hazard/failure/damage limits equal to 1. Next, the local maxima of the 1D biosystem key component are merged into one temporally‐increasing synthetic vector $R\left(t\right)\equiv \vec{R}=\left(R_{1},R_{2},\ldots ,R_{N}\right)$ in accordance with the corresponding merged temporal biosystem vector $t_{1}<\cdots <t_{N}$, $N\leq N_{X}+N_{Y}+{\;N}_{Z}+\ldots$. Each bio or public health system's principal component's local maxima $R_{j}$ are actually encountered by the bio‐system's principal components, corresponding to either X(t) or Y(t), or either Z(t) or other biosystem's critical components. The $\vec{R}$‐vector of the assembled synthetic biosystem's $\vec{R}$‐vector has 0 data loss, as shown in Figure 2.

The synthetic biosystem vector $\vec{R}$, along with its temporally‐increasing occurrence temporal instants $t_{1}<\cdots <t_{N}$, is now fully introduced.

## 3 RESULTS

Prediction of HIV‐type epidemics has been a long time within the focus of the epidemiological community, within fields of virology, and math biology. It is known that public health system bio‐dynamics can be viewed through multidimensional, highly nonlinear, spatially inter‐correlated biological or public health systems, which is quite challenging to foresee. Previous research studies have utilized a variety of statistical approaches to model the PDF of raw HIV clinical case numbers. The current section aims to illustrate the practical efficiency of the above‐described risk assessment methodology by means of applying it to a real‐life HIV raw clinical dataset, represented as annually recorded raw timeseries, for all world's nations. The raw clinical dataset utilized in the current section was obtained from the official public website^26^; this website provided HIV death rates for each country in the world between 1990 and 2019. The patient's death rate numbers from 195 different world countries have been chosen as biosystem principal components X,Y,Z,… thus constituting an example of a 195D (i.e., 195‐Dimensional) dynamic biosystem. To combine all 195 measured/observed time series X,Y,Z,… the following uniform scaling was performed, following Equation (2), resulting in all 195 biosystem critical components being nondimensional, having identical failure/hazard limits equal to 1. Hazard/failure/risk/damage limits/thresholds $\eta_{X},\eta_{Y},\eta_{Z},\ldots$, or epidemic thresholds, which are not always the obvious choice. For different countries, the simplest obvious choice would be to set hazard/failure/risk/damage limits equal to the corresponding country/nation total death number, in % (percent) to the national population, making X,Y,Z,… equal to the% annual death rate, per country. All biosystems' principal component's local maxima from all 195 measured time‐series have been merged into 1 single timeseries, by keeping them in temporally increasing order $\vec{R}=\left(\max\left\{X_{1},Y_{1},Z_{1},\ldots \right\},\ldots ,\max\left\{X_{N},Y_{N},Z_{N},\ldots \right\}\right)$ with the whole biosystem's vector$\;\vec{R}\;$being sorted, according to increasing temporal order of occurrence of these biosystem critical components' local maxima.^35^, ^36^, ^37^, ^38^, ^39^, ^40^, ^41^, ^42^, ^43^

**Figure 3:**
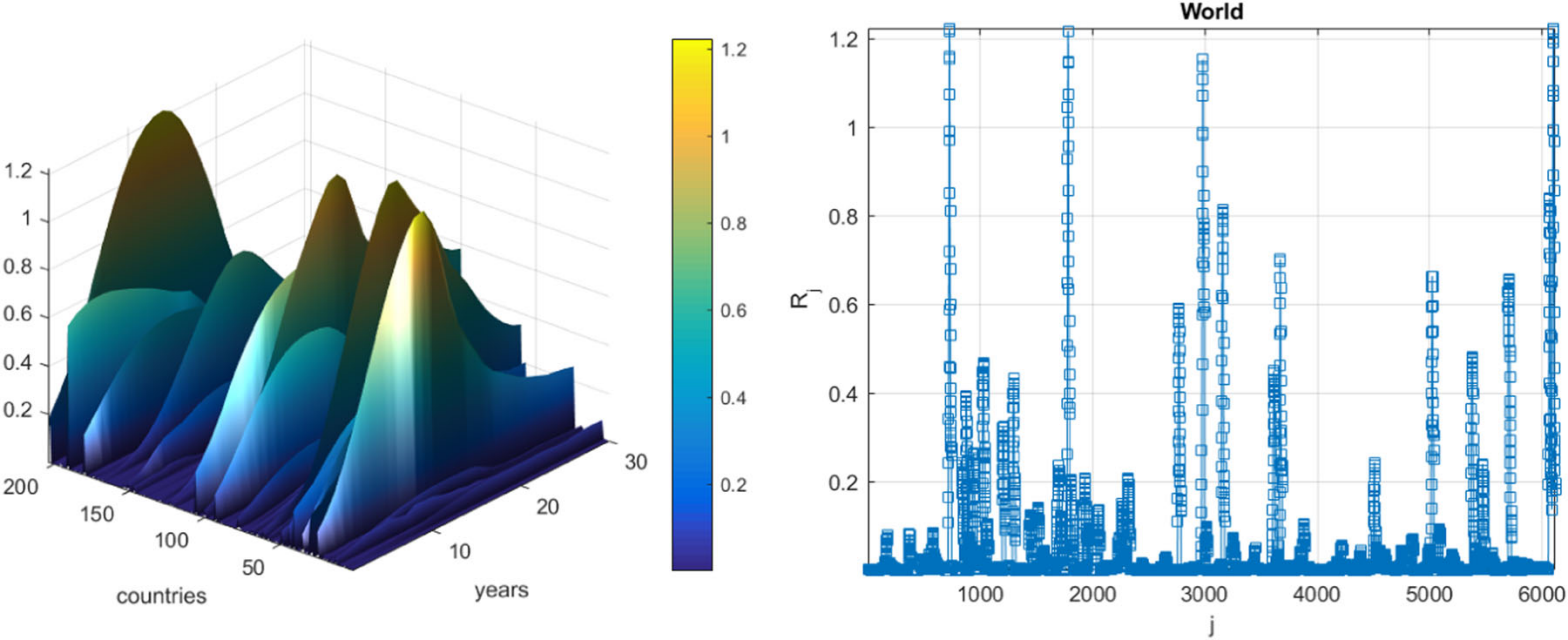
Annual recorded deathrates in %. Left: As 2D surface; Right: As synthetic vector $\vec{R}$. Scaled by Eq. (12) in % of the corresponding country's population.

Figure 3 on the right presents the numbers of newly annually recorded HIV‐related deaths, as synthetic bio‐vector $\vec{R}$, consisting of the combined annual deathrate per each world's country. Vector $\vec{R}$ comprises different regional/national components with different epidemic backgrounds. The index j is the running integer index of the local component maxima recorded in an increasing temporal sequence.^44^, ^45^, ^46^, ^47^, ^48^, ^49^, ^50^

**Figure 4:**
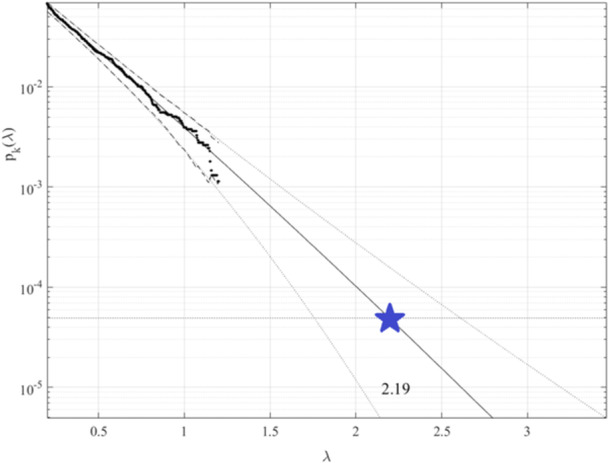
Deathrate's prediction. $p_{k}\left(\lambda \right)$ extrapolation towards target critical level (100‐years return level, marked with blue star) in % to national population. Extrapolated 95% CI (i.e., Confidence Interval), indicated by dotted lines, horizontal axis shows % of national populations.

Figure 4 shows the extrapolation of annual death rates (% of deaths from HIV‐related deaths per country) towards the epidemic outbreak level with a 100‐year return period, marked by a horizontal dotted line. λ=0.2% cut‐on value has been utilized, % of local populations on horizontal axis. Dotted lines mark extrapolated 95% CI, with p(λ) being directly related to the target hazard/failure/damage probability/risk 1−P from Equation (1).^51^, ^52^, ^53^, ^54^, ^55^, ^56^ Hence, the biosystem hazard/failure/damage risk/probability $1-P\approx {1-P}_{k}\left(1\right)$ can be accurately assessed. N equals the total number of biosystem critical components' local maxima within a unified biosystem's 1D vector$\;\vec{R}$.^57^, ^58^, ^59^, ^60^, ^61^ Conditioning memory depth number k=3 had been found to be sufficient, owing to the reasonable convergence occurrence, with respect to k.^62^ Figure 4 exhibits fairly narrow 95% CI; the latter is a distinctive advantage of advocated bio‐reliability method.^62^, ^63^, ^64^, ^65^, ^66^, ^67^, ^68^ To validate the proposed methodology, 2 smaller datasets were used to obtain predictions for identical risk and hazard levels of interest, as shown in Figure 4. A smaller dataset was obtained from the original raw clinical dataset by sampling each 2nd consecutive datapoint. Predicted value of λ, Based on the reduced dataset, has been found to lie within 95% CI based on the full dataset, indicated in Figure 4. SODP (i.e., 2nd order difference plot) originates from the Poincare‐type plot; SODP reflects the biostatistical pattern of consecutive $\vec{R}\;$vector component differences in the raw time‐series dataset.

**Figure 5:**
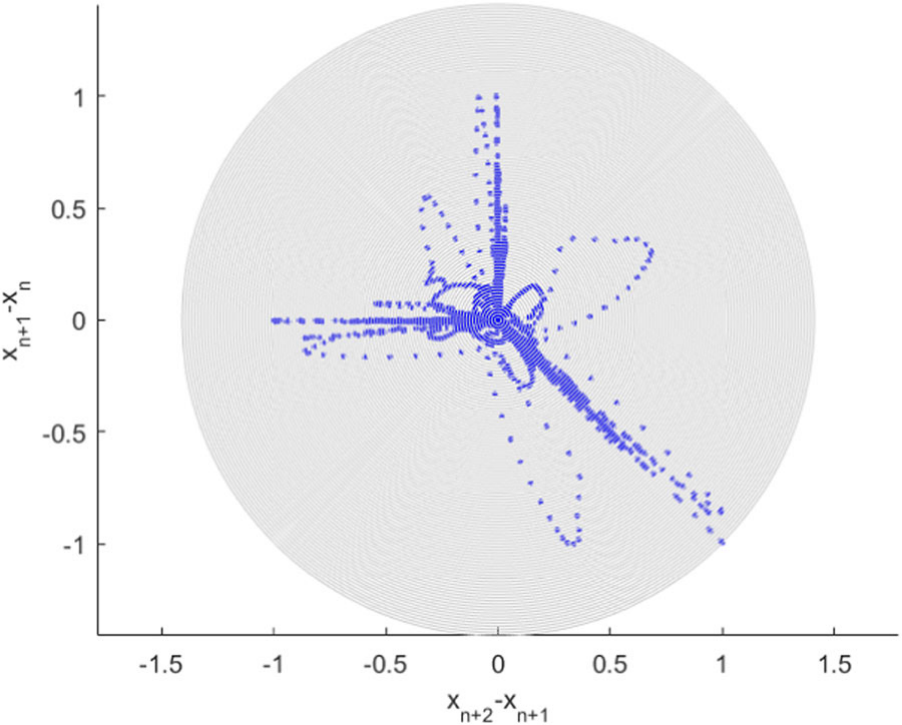
HIV deathrate global statistics. 2nd order SODP plot.

Figure 5 presents 2nd order SODP plot; such types of plots may be utilized for the purpose of the underlying raw dataset pattern recognition, as well as a comparison with similar clinical datasets, for example, for entropy AI (i.e., Artificial Intelligence) recognition approaches.^62^ Figure 5 presents an unnatural three‐spiky data pattern, which may hint at data manipulation. EVT is by definition asymptotic and 1D, while the current study introduces MDOF along with the sub‐asymptotic approach.^69^, ^70^, ^71^, ^72^, ^73^, ^74^, ^75^, ^76^, ^77^, ^78^, ^79^, ^80^

## 4 DISCUSSION

Most countries in the world have HIV death rates of less than ten deaths per 100,000, and often even lower, namely below five per 100,000. Europe's death rate is less than one per 100,000. Predicted HIV deathrates in any world's country in years to come, the next 100‐years have been found about 2.2%. While the advocated methodology is novel, it has clear advantages of utilizing the underlying raw measured dataset quite efficiently because of the method's ability to treat health biosystem multi‐dimensionality as well as to perform accurate extrapolations based on quite limited raw underlying clinical datasets. Note that the predicted nondimensional λ level, indicated with the star in Figure 4, represents the risk/probability of epidemic outbreaks in any world's country, within the near future.^81^, ^82^, ^83^, ^84^, ^85^, ^86^

Biosystems with high dimensionality, along with complex inter‐correlations between various critical components of the biosystem, are not easily handled by traditional public health bio‐risk assessment approaches that deal with raw clinically observed time‐series.^87^, ^88^, ^89^, ^90^, ^91^, ^92^, ^93^ The capacity to examine the reliability of a high‐dimensional, nonlinear dynamic biosystem is the main benefit of the advocated approach. Despite its overall simplicity, the present study offers a multidimensional novel modeling strategy along with a methodological avenue suitable for forecasting future excessive HIV deathrates. Proper settings of epidemiological alarm/risk limits (risk/failure/hazard/damage limits) for each nation/country are briefly discussed.^94^, ^95^, ^96^, ^97^, ^98^, ^99^, ^100^, ^101^, ^102^

## 5 CONCLUSIONS

The current study examined recorded HIV deathrates from all world countries/nations, constituting an example of a 195‐dimensional dynamic biosystem, observed within three recent decades 1990–2020. A novel bio‐risk assessment methodology has been applied to HIV annual death numbers, constituting multidimensional biosystems in real‐time. The theoretical reasoning behind the advocated methodology is provided in brief detail. The use of extensive clinical measurements or direct MCS for dynamic biological or public‐health systems bio‐risk assessment analysis is quite attractive; however, the complexity of the bio and public health dynamic system, along with its high dimensionality, requires the development of robust, novel, and accurate techniques that deal efficiently with even limited raw clinical datasets.

The main conclusion of this investigation is that the world's public health biosystem under local epidemiological and environmental conditions has been well managed. current study assessed annual deathrate 100‐year return period risk level, found to be ~2%. Hence, under the current national health management conditions, given wide range of effective measures of prevention that would stop people from getting HIV, the threat of HIV to the world's health will remain to stay low. The main objective of this study is to apply a general‐purpose, reliable, and simple approach to a multi‐dimensional bio‐risk assessment approach. The approach described in the current study has already been validated by applications to a broad variety of contemporary engineering models; however, only for one‐dimensional system components, accurate predictions have been obtained. Public health system's dynamics in the form of timeseries can be obtained either via clinical measurements or MCS. The proposed methodology yields a narrow confidence interval. Hence, the recommended methodology may be useful for a wide range of biological or public health risk assessment investigations governed by nonlinear dynamic biological or public health systems. The present HIV case does not limit the potential applicability of the advocated methodology.

## CONFLICT OF INTEREST STATEMENT

The author declares no conflicts of interest.

## DATA AND CODE AVAILABILITY

The datasets analyzed during the current study are available online at https://ourworldindata.org/hiv-aids, for code see https://github.com/OlegGaidai/HIV-deathrate-prediction.

## ETHICAL APPROVAL AND CONSENT TO PARTICIPATE

Ethical approval had not been required as no patients were involved.

## CONSENT FOR PUBLICATION

The author agreed.

## SOFTWARE AVAILABILITY

The source code, demo, user manual, and examples used for extrapolation are available at https://github.com/OlegGaidai/HIV-deathrate-prediction.
